# Ultraviolet Luminescence of ZnO Whiskers, Nanowalls, Multipods, and Ceramics as Potential Materials for Fast Scintillators

**DOI:** 10.3390/ma14082001

**Published:** 2021-04-16

**Authors:** Ivan D. Venevtsev, Andrey P. Tarasov, Arsen E. Muslimov, Elena I. Gorokhova, Ludmila A. Zadorozhnaya, Piotr A. Rodnyi, Vladimir M. Kanevsky

**Affiliations:** 1 Department of Physics, Peter the Great St. Petersburg Polytechnic University, 195251 St. Petersburg, Russia; venevtsev.ivan@gmail.com (I.D.V.); piotr_rodnyi@mail.ru (P.A.R.); 2 Federal Scientific Research Centre “Crystallography and Photonics” of Russian Academy of Sciences, Shubnikov Institute of Crystallography, 119333 Moscow, Russia; tarandrew17@gmail.com (A.P.T.); vpzadorozhny@mail.ru (L.A.Z.); kanev@crys.ras.ru (V.M.K.); 3 S.I. Vavilov State Optical Institute, 36 Babushkina St., 192171 St. Petersburg, Russia; e.gorokhova@rambler.ru

**Keywords:** zinc oxide, fast scintillators, total optical transmittance, radioluminescence, X-ray induced luminescence, photoluminescence, near-band-edge luminescence, ceramics, nanowalls, whiskers, multipods

## Abstract

The presented work is dedicated to the study and comparison of scintillating properties of zinc oxide samples prepared in different morphologies: whiskers, nanowalls, multipods, and ceramics. It was shown that total transmittance, photo- and radioluminescence spectra, and radioluminescence kinetics can vary significantly depending on sample structure and preparation conditions. The highest total transmittance was registered for ZnO ceramics (>50% at 0.5 mm thickness). Differences in the transmittance of whiskers, nanowalls, and multipods can be attributed to their shape and thickness which affects the amount of light refraction and scattering. The study of radioluminescence demonstrated that all samples, except undoped ceramics and air annealed whiskers, have predominantly fast luminescence with a decay time <1 ns. High transmittance of ceramics opens the way for their use in the registration of high energy X-ray and gamma radiation, where a large volume of scintillators is required. In cases, where large scintillator thickness is not a necessity, one may prefer to use other ZnO structures, such as ensembles of whiskers and nanowalls. Studies of near-band-edge luminescence components at low temperatures showed that the structure is quite similar in all samples except Ga doped ceramics.

## 1. Introduction

The development of modern accelerators follows the path of increasing energies and higher luminosity, thus giving rise to the demand for detectors with improved characteristics and higher reliability. Scintillation counters, in general, meet these requirements. However, fabricating a scintillator with a high light output and a short response time is a challenge. In this regard, zinc oxide (ZnO) is one of the most promising materials. ZnO is a wide direct band gap semiconductor with *E_g_* ~ 3.3 eV at room temperature (RT) [1]. The high exciton binding energy (~60 meV) allows for the observance of a high-intensity excitonic luminescence at and above room temperature [1]. ZnO has good radiation hardness [2], however, due to its low density, its use in the registration of high-energy X-ray and gamma radiation can be restricted. Despite this, various authors have reported on ways to improve the spatial and temporal resolution of scintillators based on ZnO [3,4]. Successful application of a ZnO-based detector for the detection of nuclear radiation is reported in [5,6].

Zinc oxide emission spectra usually contain two luminescence components: ultraviolet emission near the edge of ZnO fundamental absorption (near-band-edge (NBE) emission) in the spectral range of 380–400 nm and green luminescence (GL) in the spectral range of 450–650 nm [1]. NBE luminescence has a characteristic lifetime in a sub-nanosecond range [7], which makes it favorable in applications requiring fast counting. Green luminescence, believed to originate from native defects in the ZnO crystal lattice [8], has a lifetime of around 1 μs and a much higher light output. Therefore, the highest scintillating efficiency should be observed for bulk ZnO single crystals [9] with a low concentration of point defects and dislocations. However, there is currently no optimal technology for the fast and cost-effective production of such crystals. Different attempts to grow them [10] only prove to be technically difficult and have a high cost. ZnO films could be an alternative to bulk ZnO crystals but their thickness of 1–2 μm only makes them usable for the detection of alpha particles [11]. With an increase in the thickness of the films, the probability of their cracking and peeling also increases due to greater stresses in the bulk of the film and at the interface with the substrate. Another suggestion was to use scintillating ZnO ceramics [12,13] or ensembles of different ZnO micro- and nanostructures. Due to a wide range of different morphologies, a multitude of synthesis methods was suggested, such as a microwave-assisted method [14], hydrothermal method [15], solvothermal method [16], etc. Most of the further research was directed toward the effective quenching of GL [17,18]. Along with that, fast NBE luminescence still plays the main role. A review by [19] on ZnO scintillation properties shows that the light yield of ZnO can vary in the range of 10–120% of the BGO light yield, depending on the doping and preparation conditions. In this regard, it is relevant to study spectral and kinetic features of NBE luminescence and compare them between different morphologies. The understanding of properties and mechanisms of NBE emission will allow for the preparation of ZnO-based scintillators with improved characteristics. This work is dedicated to the study of optical and scintillating properties of ensembles of ZnO whiskers, nanowalls, multipods, and undoped and Ga-doped ZnO ceramics. To show the effect of morphology we compare total transmittance, NBE emission intensity, and decay kinetics of those structures. Photoluminescence (PL) spectroscopy was used along with radioluminescence (RL) measurements for better comparison.

## 2. Materials and Methods

The synthesis of ZnO structures of various morphologies was carried out using the previously developed technique of gas-phase synthesis from basic elements in a horizontal flow reactor [20]. A quartz boat with a high purity (99.99%) Zn powder (CAS number 7440-66-6) was placed at the sealed end of the quartz ampoule. A wide slit on the upper wall of the ampoule, opposite to where the substrates were placed, played the role of a diaphragm separating the gas flows of Ar (CAS number 7440-37-1) and O_2_ (CAS number 7782-44-7). Single-crystal sapphire plates 10 mm × 10 mm in size with double-sided polishing were used as substrates. An ampoule with a Zn source and substrates was installed in a flow-through reactor with two heating zones so that Zn was in one zone (evaporation zone), and the substrate was in another zone (growth zone). The reactor was evacuated using a fore vacuum pump for an hour (the residual pressure was ~20 Pa). Then, Ar was fed into the chamber and the substrates were heated to a temperature of 550–580 °C. At the next stage, the temperature in the evaporation zone was raised, which was also varied in various experiments in the range of 620–650 °C. After reaching the operating mode, oxygen (high purity grade) was fed into the reactor in the ratio Ar/O_2_ ~ 9/1. The duration of the synthesis varied in the range of 5–30 min. A fairly wide range of changes in the synthesis conditions is required to find the optimal growth parameters for obtaining oriented, high-quality ZnO nanocrystallite and microcrystallite arrays.

Self-catalytic and catalytic growth modes were used. In the first case, a deposited zinc film played the role of a metal catalyst. In the second case, a silver film was preliminarily deposited on the substrate according to the following scheme. A thin silver (CAS number 7440-22-4) layer 12 nm thick was deposited on one half of the substrate (region I). The second half of the substrate (region II) was without preliminary preparation. The deposition was carried out by the thermal evaporation method on a VN-2000 vacuum spraying unit (Akadempribor, Ukraine) and the layer thickness was monitored using a KITP-5 quartz thickness gauge (Bonfon, Belarus).

This paper presents the results of the analysis of whiskers, nanowalls, and multipods which were obtained within 20 min. Several samples of each type were generated in order to test reproducibility. Overall, the characteristics of samples between batches varied less than 15%.

ZnO and ZnO:Ga ceramics have been prepared by the hot uniaxial pressing technique. This technique has been used earlier to produce highly transparent polycrystalline ZnO:Zn [21] ceramic bodies, making them a candidate for application as scintillators. Ga was added by the mechanical admixture of Ga_2_O_3_ (CAS number 12024-21-4) into the initial ZnO powder (CAS number 1314-13-2). Ceramic samples have been obtained in the form of discs with 0.5 mm thickness.

Microscopic studies were carried out using a JCM-6000 Neoscope 2 (JEOL, Tokio, Japan) scanning electron microscope (SEM). The average layer thickness was determined by examining the cross-sections of the samples with the electron microscopy method.

Total transmittance spectra were obtained using a SPECORD 200 PLUS (Analytik Jena GmbH, Jena, Germany) double-beam spectrophotometer equipped with an integrating sphere.

RL spectra were measured under continuous X-ray excitation (40 kV, 10 mA) from a tube with a tungsten anode. The measurement part of the setup contained an MDR-2 monochromator (LOMO Photonika, St. Petersburg, Russia) and a H8259-01 photon counting system (Hamamatsu Photonics, Shizuoka, Japan). The obtained spectra were corrected taking into account the sensitivity of the photodetector and the monochromator. In the measurements, we used “reflection” geometry with an angle of 90°; the sample was installed at an angle of 45° to the excitation source and the measurement path. RL spectra were recorded at room temperature (RT) and a temperature of ~80 K. The samples were cooled using a vacuum cryostat. Investigations of the RL kinetics were carried out in an integral mode, i.e., without isolating a certain spectral range, with pulsed X-ray excitation using the method of time-correlated single photon counting using the setup described in [21].

The PL of ZnO structures was excited by a frequency-tripled Nd:YAG laser (355 nm) with repetition rate of 15 Hz and pulse duration of 10 ns (LOTIS TII, Minsk, Belarus) and recorded using an MDR-206 monochromator (LOMO Photonika, St. Petersburg, Russia) coupled with a Peltier-cooled CCD camera (Videoscan, St. Petersburg, Russia). PL measurements were performed at RT.

## 3. Results

**Figure 1:**
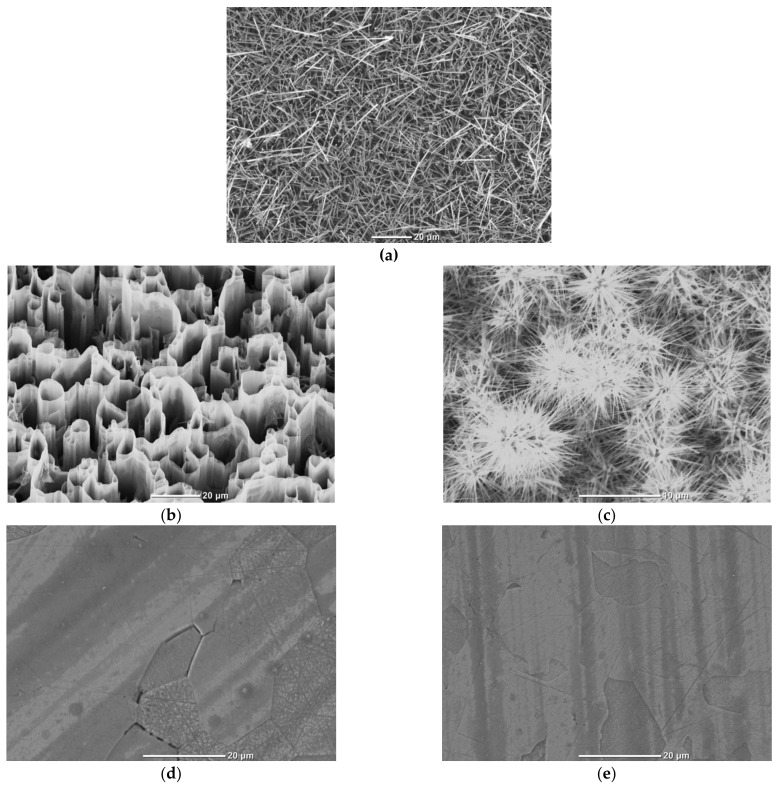
SEM-images of studied ZnO samples: (**a**) whiskers; (**b**) nanowalls; (**c**) multipods; (**d**) undoped ceramics; (**e**) 0.075 wt.% Ga doped ceramics.

In the case of using self-catalytic synthesis on a sapphire substrate at a temperature of 580 °C, cylindrical whiskers with a diameter of 0.5–1.0 μm (Figure 1a) and a height of up to 50 μm were grown. An ensemble of whiskers is characterized by a uniform distribution over the substrate surface and, at the same time, a strong misorientation of crystallites relative to each other. In the region of the base of the whiskers, a polycrystalline layer containing an excess amount of zinc was formed. The formation of this layer is explained by the competition between two different mechanisms of crystallization of ZnO: microrods grow according to the vapor-liquid-crystal (VLC) mechanism, and the formation of a polycrystalline layer proceeds according to the direct vapor-crystal (VC) mechanism. A feature of gas-phase processes involving chemical reactions is their high sensitivity to heterogeneities on substrates. Therefore, if a microcrystallite is formed on the surface of the substrate by means of vapor deposition, then after reaching dimensions of 5–10 nm it will grow at an ever-increasing rate and its mobility will rapidly decrease, practically excluding the possibility of oriented growth.

In [22], catalytic synthesis using a thin Al metal layer previously deposited on a substrate was investigated. When the substrate is heated up to the beginning of the growth process, a liquid metal sublayer is formed, which decomposes into isolated island-shaped clusters (drops), which are the main centers of nucleation, orientation, and subsequent prevailing growth of ZnO nanocrystallites by the VLC mechanism. In this work, the heating and decomposition of a thin silver film with the subsequent formation of quasi-liquid metal islands on the substrate (region I) occurs directly in the growth zone (when the substrate is heated to 580 °C for 20 min) in an Ar flow prior to the onset of the crystallization process.

Microscopic images of the grown ZnO arrays on a substrate with an Ag sublayer are shown in Figure 1b (region I) and Figure 1c (region II). Comparison of the morphologies of the synthesized structures clearly demonstrated the role of the Ag sublayer as a catalyst for the directed growth of ZnO structures. The ZnO structures (Figure 1b) grown in the silver-coated region (region I) had a perfect and developed morphology, representing vertical labyrinths consisting of ordered branched nanowalls oriented perpendicular to the substrate. The thickness of the nanowalls was less than 100 nm, the height was up to 20 µm, and the width of the labyrinths was up to 20 µm. The geometry of the synthesized structure is similar to the previously obtained nanowalls described in [23].

Figure 1c shows the morphology of the ZnO structure grown on a substrate without a silver layer (region II). It consisted mainly of multiple groups of mutually disordered needle-like crystals grown from one center (multipods). The spatial arrangement of microcrystals was close to chaotic. It can be assumed that the formation of such a structure is explained by the competition of two different mechanisms of ZnO crystallization: (i) the formation of a polycrystalline layer proceeds according to the direct VC mechanism; (ii) stellate microrods grow from large drops of Zn according to the “self-catalytic” VLC mechanism. By analogy with whiskers, the polycrystalline layer can contain Zn in excess. The sizes of ZnO multipods are 10–50 μm, the diameter of the needles is up to three microns. Each multipod is an independent crystal growing in the wurtzite modification. The dense arrangement, layering of polycrystalline structures in the region of the substrate, is probably due to the high supersaturation of the reaction vapors. It should be noted that region II was closest to the zinc source, which created favorable conditions for the formation of ZnO multipods.

The microstructure of undoped ceramics (Figure 1d) was represented by grains having a shape close to isometric, with sizes in the range of 10–35 μm, which significantly exceeded the particle size of the initial powder due to the intense recrystallization processes. The microstructure of ZnO:Ga ceramics (Figure 1e) is composed of grains of indefinite shape without clear faceting with sinuous boundaries, as well as grains whose shape approaches isometric. The grain size varied mainly within 5–20 µm. All ceramic samples were about 0.5 mm thick.

**Figure 2:**
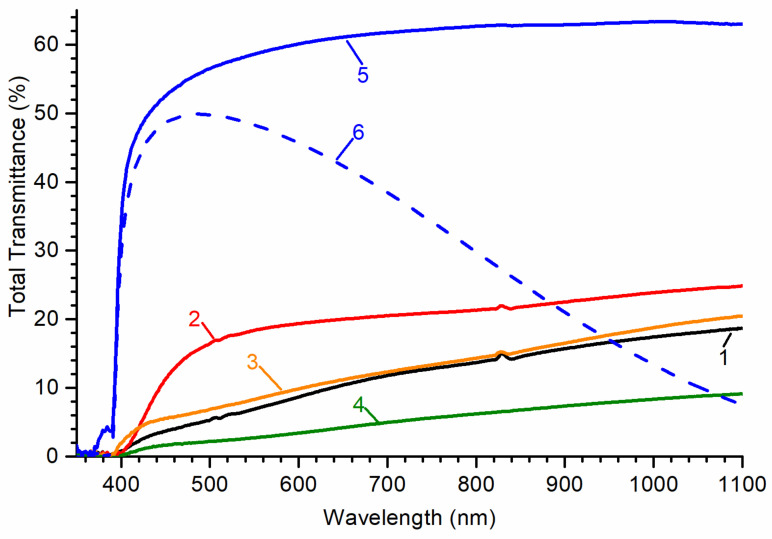
Total transmittance spectra of studied ZnO samples: 1—whiskers; 2—whiskers after annealing in air; 3—nanowalls; 4—multipods; 5—undoped ceramics; 6—ceramics doped with 0.075 wt.% of Ga.

The next step was to measure the total transmittance spectra in the range of 350–1100 nm (Figure 2). It can be seen that the samples strongly differ in the position of the absorption edge, shape, and maximum value of total transmittance. At the same time, when comparing samples, it is necessary to take into account the differences in their thickness.

In the case of the ensemble of ZnO whiskers, a rough estimate of the position of the absorption edge gave, respectively, 390 and 398 nm for the sample before and after annealing in air. In general, transmittance spectra for ZnO whiskers can be conditionally divided into three parts, which differ noticeably in the slope of the curve: from the transmission edge to 420 nm, 420–640 nm, and 640–1100 nm. After annealing in air, the transmittance increased significantly. At the same time, the characteristic region 420–640 nm disappeared.

The absorption edge of ZnO nanowalls and multipods is at 391 and 397 nm, respectively. Their transmittance spectra also contain an intermediate section with a different slope, but it is less pronounced in comparison with whiskers. The worst transmittance in the visible region of the spectrum is possessed by an array of multipods.

For undoped ZnO ceramics and ZnO:Ga ceramics, the position of the absorption edge is very close and is about 390 nm. The edge slope is much sharper compared to the rest of the samples. Nonzero transmittance in the 370–390 nm range for the undoped sample is an artifact of the setup (GL excited by the beam reaches the photodetector since there is no monochromator in the registration path). The transmittance of the undoped sample reached a maximum of ~63% in the 800–1100 nm region. The Ga doped sample had a maximum transmittance of ~50% at 485 nm after which the transmittance rapidly decreased.

**Figure 3:**
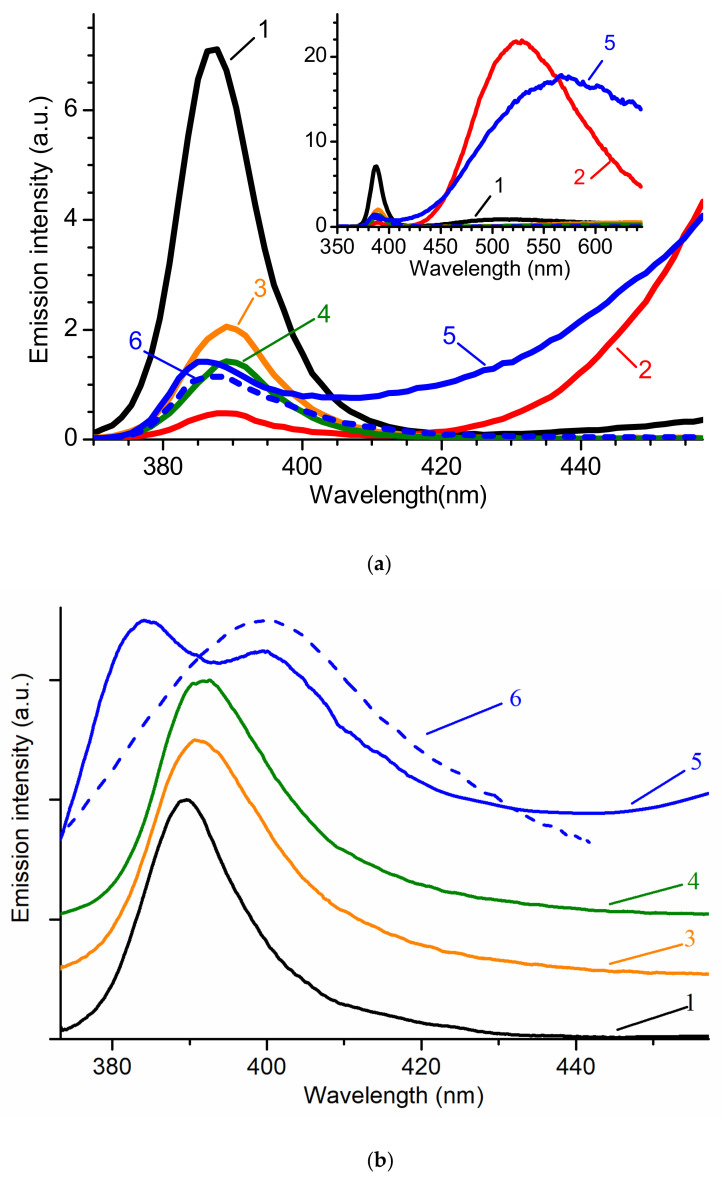
RT luminescence spectra of studied ZnO samples under X-ray (**a**) and photo- (**b**) excitation for: 1—whiskers; 2—whiskers after annealing in air; 3—nanowalls; 4—multipods; 5—undoped ceramics; 6—ZnO ceramics doped with 0.075 wt.% of Ga. PL spectra were normalized at maximum and vertically shifted for clarity.

Figure 3a shows the RL spectra of the samples. For ease of comparison, their relative intensities have been retained. The inset in Figure 3a shows the long-wavelength part of the luminescence spectrum.

Under X-ray excitation, most of the samples exhibited pronounced NBE luminescence. In the long-wavelength region of some samples, there is also a GL band. It is most noticeable in undoped ZnO ceramics and an ensemble of whiskers after annealing in air. NBE emission in untreated whiskers reached a maximum of 387 nm. The band intensity was the highest among the studied samples. Heat treatment in air degrades the characteristics of the sample. The integrated RL intensity decreased by more than a factor of 10 and the maximum was shifted to 388.5 nm. In this case, the GL band appeared with a maximum at ~525 nm and had a ~300 times higher integrated intensity in comparison with NBE emission.

Nanowalls and multipods also exhibited rather intense NBE luminescence with a maximum at 389 nm and 389.5 nm, respectively. GL in these samples is not registered, and the integrated emission intensity is comparable to the NBE luminescence intensity of ceramics. In this case, the RL maximum of ZnO:Ga ceramics is at 387 nm, and the maximum of undoped ZnO ceramics is at 385.5 nm. In undoped ZnO, a strong GL band was observed, the intensity of which was comparable with that of the GL band for annealed whiskers. Meanwhile, it had a different position of the maximum (~560 nm) and was comparatively wider. The introduction of Ga led to the quenching of the GL band, leaving the NBE emission intensity almost unchanged.

In Figure 3b, the PL spectra are shown for the array of whiskers at an excitation fluence of 0.1 MW/cm^2^, as well as nanowalls, multipods, undoped ceramics, and ZnO:Ga ceramics at a fluence of ~0.6 MW/cm^2^. The maxima of the PL spectra of whiskers, nanowalls, and multipods were observed at 389.5 nm, 391 nm, and 391.5 nm, respectively. The NBE luminescence of undoped ceramics consisted of two components with maxima at 384.5 nm and 399 nm, while ZnO:Ga ceramics consisted of only one component at 399 nm. The difference in the excitation fluence is due to the different luminescence intensities. For the same reason, the data on the graph are normalized and vertically shifted for clarity of comparison.

Under photoexcitation, GL was recorded only for undoped ZnO ceramics (not shown). In this case, the GL band had its maximum at ~515 nm and exceeded the NBE emission by only ~two times (in terms of the integral intensity).

Differences in the shape and position of NBE emission bands under different types of excitation could be due to the peculiarities of their spectral composition. At RT, recorded spectra may represent a combination of several components that are poorly resolved with each other due to thermal broadening of the bands or other reasons. Taking into account that the penetration depth of X-ray radiation is several orders of magnitude greater than that of optical radiation with photon energy greater than the band gap, it can be assumed that, depending on the type of excitation, the relative intensity of these components changes, which causes a shift in the spectrum. A large number of defects or nonradiative recombination centers at grain boundaries can also be the reason. For example, defects can form during the polishing of ceramics.

**Figure 4:**
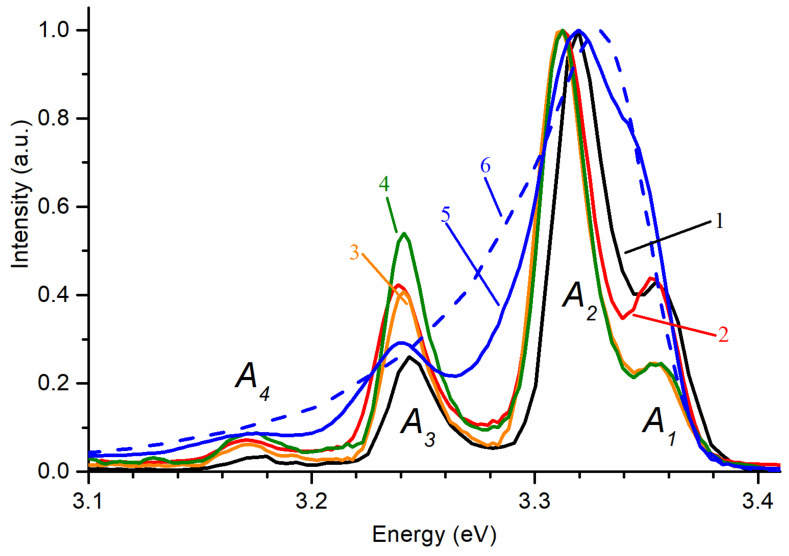
RL spectra at 80 K in the spectral range of NBE emission of ZnO samples: 1—whiskers; 2—air annealed whiskers; 3—nanowalls; 4—multipods; 5—undoped ceramics; 6—ZnO:Ga ceramics. Spectra are normalized at maximum.

Additional information about the specific features of the NBE emission band can be obtained by studying its structure at low temperatures. Figure 4 shows the low-temperature RL spectra of the studied samples. For the convenience of interpretation of the spectral components, spectra were converted to an energy scale. Four luminescence bands are visually registered, which are indicated in the order of the decreasing of their energy as *A*_1_, *A*_2_, *A*_3_, and *A*_4_ in Figure 4. The *A*_1_ and *A*_2_ bands are not resolved in undoped ZnO ceramics, while in ZnO:Ga ceramics all bands merged into one asymmetric peak.

The poorer resolution of the recorded bands in the NBE emission spectra of ZnO ceramics could be caused by their characteristic band bending near the grain boundaries [24]. Such a bend occupies a spatial region near the surface of ceramics grains, comparable in size to the absorption depth of optical radiation. As a consequence, its presence can be reflected in the PL spectra, leading to additional differences from RL.

In the case of ZnO whiskers, annealed whiskers, nanowalls, and multipods, the *A_1_* band visually has a maximum at 3.355 eV, 3.353 eV, 3.353 eV, and 3.354 eV, respectively. The precise determination of the *A*_1_ position is hindered by the overlapping *A*_2_ band, which has a higher intensity (in unannealed whiskers, it was shifted to the right, which could also shift the visible position of the *A*_1_ maximum). In accordance with the literature, the *A*_1_ band corresponds to the emission of donor-bound excitons [25]. The position of the intensity maximum of the *A*_2_ band in the samples differs most strongly. Namely, it is ~3.319 and 3.313 eV for unannealed and annealed whiskers, and 3.311 eV, 3.312 eV, 3.320 eV, for nanowalls, multipods, and undoped ceramics, respectively. The *A*_3_ and *A*_4_ bands are separated from the *A*_2_ band by an average of 74 meV and 143 meV for whiskers, 70 and 140 meV for nanowalls and multipods, and by 80 meV and 150 meV for undoped ZnO ceramics, respectively. These values are close to the single and double energies of the longitudinal optical (LO) phonon in ZnO (72 meV [26]), which suggests that *A*_3_ and *A*_4_ are phonon replicas of *A*_2_. Taking into account the relatively high intensity of the *A*_2_ band, according to various data, it can be associated with donor-acceptor pairs (DAP) [27,28], or be the first phonon replica of the free exciton recombination emission band (FX-LO) [25], or be associated with surface defects [29,30,31]. The latter one seems more appropriate since it could explain its displacement during the annealing of the whisker array. The reason for the high intensity of this band in the arrays of nanocrystallites can be a stronger relative contribution of the surface and the polycrystalline layer in comparison with ceramics, where the *A*_2_ band is comparable in intensity with *A*_1_.

**Figure 5:**
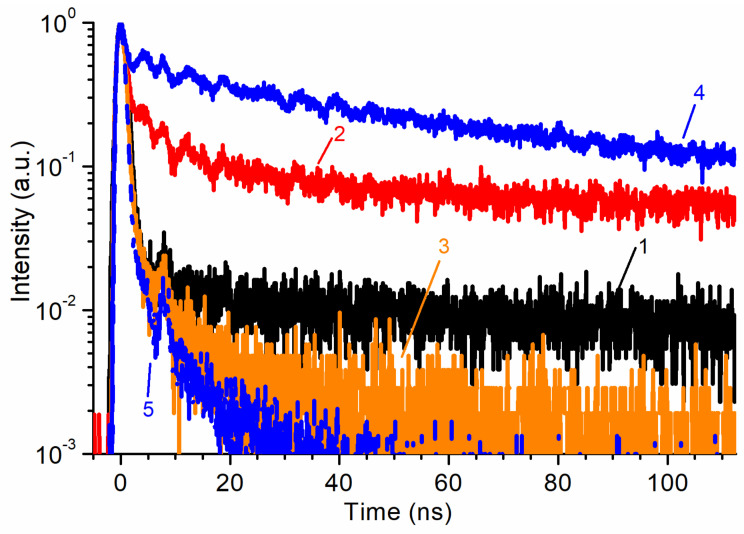
RL decay kinetics of studied ZnO samples: 1—whiskers; 2—whiskers annealed in air; 3—nanowalls; 4—undoped ceramics; 5—doped with 0.075 wt.% Ga ceramics.

The RL decay kinetics of some samples in the range of 0–110 ns are shown in Figure 5. The sample of multipods is not shown due to the very low signal intensity recorded using our instrumentation. The presence of two luminescence components is clearly visible. The fast component corresponding to NBE emission is dominant in unannealed whiskers, nanowalls, and ZnO:Ga ceramics. This corresponds to the spectral pattern in Figure 3a. The fast component fits entirely into the 0–5 ns range and has a decay time of no more than 1 ns, taking into account the excitation pulse width of ~1 ns. The slow component is present in some quantity in all samples. It is the least pronounced in ZnO:Ga ceramics. In undoped ceramics and in a sample of ZnO whiskers annealed in air, the slow component has the highest relative intensity.

## 4. Discussion

Depending on the detector geometry, the transparency of the scintillator can play a different role. Among the studied samples, this parameter varies the most. Obviously, if there is a need for a thick scintillator (for example, when constructing gamma spectrometers or ionization calorimeters), a high transmittance, especially in the immediate vicinity of the absorption edge, will be one of the main criteria. On the other hand, in the manufacture of thin scintillation screens, this parameter can become secondary.

The worst transmittance, both in terms of the maximum value and the location of the absorption edge, was registered in ZnO multipods. Unannealed whiskers have somewhat better characteristics. In annealed whiskers, despite the increased transmittance, the absorption edge is shifted by 8 nm to longer wavelengths. For nanowalls and both ceramics, the absorption edge is located at 391 nm and 390 nm, respectively which is the best result among all samples. Ceramics, however, have several times higher transmittance at a greater thickness, which makes its choice more preferable when registering high-energy radiation or radiation with a longer mean free path in a substance.

Such a low transmittance value in multipods may be a consequence of their morphology. The presence of a large number of “spikes” leads to a significant scattering of light on their surfaces. In the presence of surface defects, a large specific area can also contribute negatively to the transmittance. Another factor may be the presence of a polycrystalline layer with an excess of zinc at the base of multipods.

Whiskers and nanowalls, most likely, have better transmittance due to a better ordering relative to the initial growth direction, although for longer whiskers, their significant misorientation is also observed in the upper layers. From the above, we can make the assumption that the transmittance spectra shown in Figure 2 can be further improved through better growth control.

An interesting feature of total transmittance spectra of multipods, whiskers, and nanowalls is the presence of the 420–640 nm region, in which a broad absorption band is most likely present. It can be associated, for example, with the presence of intrinsic defects in the depth of the sample, the concentration of which depends on the structure of the sample and the method of its synthesis. This fact can be confirmed by the disappearance of this band as a result of annealing in air. One can assume that this band is associated either with oxygen vacancies or with the presence of an excess of zinc in the polycrystalline layer near the substrate.

Total transmittance of both ZnO ceramics starts at 390 nm. However, in contrast to the undoped sample, the transmittance of Ga doped ceramics begins to gradually decrease after 490 nm. The decrease in transmittance in the long-wavelength region is a consequence of a free carrier absorption, which is usually observed in ZnO doped with a donor impurity.

When comparing RL spectra, one can immediately discard from consideration undoped ceramics and the annealed array of whiskers due to the presence of an intense GL band. The highest NBE emission intensity is exhibited by the unannealed ensemble of whiskers. ZnO nanowalls, multipods, and ZnO:Ga ceramics demonstrate lower intensities by 3.5, ~5, and ~6 times, respectively. In the case of ZnO ceramics, suppression of the slow component was achieved by introducing 0.075 wt.% Ga, which is the standard method. An attempt to improve the properties of whiskers by annealing did not lead to a positive result. Perhaps a better result could be achieved by annealing an array of whiskers in a reducing atmosphere.

**Figure 6:**
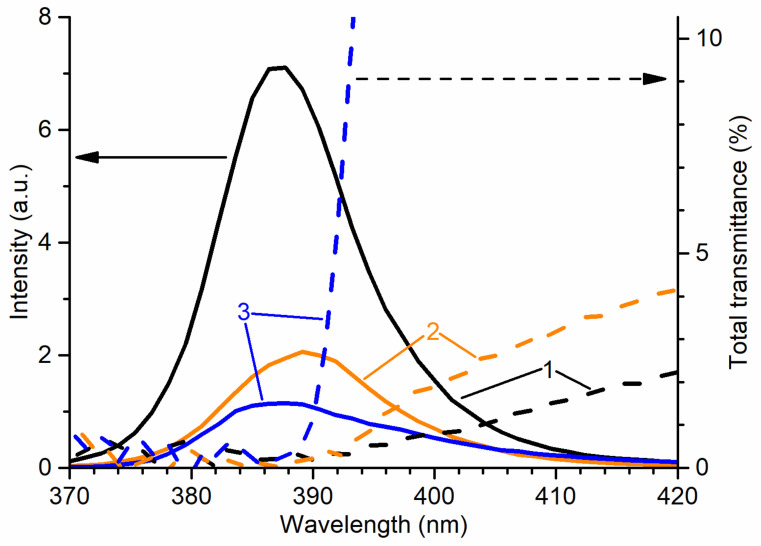
Normalized RL spectra and total transmittance spectra of 1—ZnO whiskers; 2—ZnO nanowalls; 3—ZnO:Ga ceramics at RT in the spectral range of NBE emission.

When comparing the properties of scintillators, it is convenient to present the RL and transmittance spectra in the same graph. A good indicator for such comparison is the overlap between the emission spectrum and the transmittance spectrum of the sample. It is worth noting that in our case, a small overlap could lead to the distortion of the visible RL spectrum, however, due to the use of the “reflection” geometry, this distortion should be relatively small. Figure 6 shows that ZnO:Ga ceramics outperform other types of structures in terms of the overlap. However, whiskers have a much higher intensity, which also makes this type of structure promising, especially if it is possible to select the correct annealing conditions to increase transmittance.

The RL kinetics shown in Figure 5, in principle, correlate with the RL spectra (Figure 3a). In samples of undoped ZnO ceramics and an annealed array of whiskers, the slow component corresponding to GL is dominant. A rapid luminescence decay, in turn, is characteristic of samples of whiskers, nanowalls, and Ga-doped ceramics. It is clearly seen from the figure that, when measuring in the integral mode, even a small contribution of GL leads to a significant slowing down of the decay, which will negatively affect the temporal resolution of the detector and limit its count rate. One of the ways out of the situation can be the use of optical filters, which will, however, lead to additional losses in intensity. Therefore, the best option would be to suppress GL directly in the sample by doping, annealing in a reducing atmosphere, or purposeful modification of the synthesis technology to achieve a more ordered growth.

## 5. Conclusions

In this work, the scintillation characteristics of ZnO whiskers, nanowalls, multipods, and ceramics were studied and compared. It was shown that, depending on the structure type and preparation conditions, the shape of the transmittance spectra, RL and PL spectra, as well as the RL decay kinetics can change significantly.

Comparison of the PL and RL spectra in the NBE region revealed the specific features of NBE emission under these two excitation conditions. Additional studies of RL at 80 K revealed the same structure of the spectra for all samples and a large contribution of, presumably, surface states to NBE emission in the case of crystallite arrays as compared to ceramics.

The best transmittance is possessed by ZnO ceramics (more than 50% at the maximum with a thickness of 0.5 mm). The worst in this sense were arrays of multipods. The difference in the transmittance of the ensembles of whiskers, nanowalls, and multipods, in addition to their different thicknesses, is mainly due to the difference in the shape of the crystals and their density, depending on which the degree of light scattering changes. In this respect, vertically aligned structures (nanowalls and whiskers) have an advantage.

The study of RL kinetics made it possible to demonstrate the domination of a fast luminescence component with a lifetime of less than 1 ns for all samples, except for undoped ceramics and an annealed array of whiskers. The slow decay of the luminescence of the latter is associated with the presence of a GL band in the spectrum.

The high transmittance of ceramics allows them to be used for detecting high-energy X-ray and gamma radiation, which require a large scintillator volume. If a large scintillator thickness is not required, preference can be given to other ZnO structures, namely arrays of whiskers and nanowalls. In particular, ZnO whiskers demonstrated the highest NBE emission intensity, and ZnO nanowalls were characterized by a smaller contribution of GL.

## Data Availability

Data Sharing is not applicable.
